# Pretreatment of cardiac progenitor cells with bradykinin attenuates H_2_O_2_-induced cell apoptosis and improves cardiac function in rats by regulating autophagy

**DOI:** 10.1186/s13287-021-02503-6

**Published:** 2021-08-05

**Authors:** Chan Wu, Xiao-Xia Zhou, Jing-Zhou Li, Hai-Feng Qiang, Yan Wang, Gang Li

**Affiliations:** Xiamen Cardiovascular Hospital, Xiamen University, Xiamen, 361015 Fujian China

**Keywords:** Bradykinin, Cardiac c-kit+ progenitor cells, Myocardial infarction, Autophagy, Apoptosis

## Abstract

**Background:**

Previous studies have demonstrated that human cardiac c-Kit^+^ progenitor cells (hCPCs) can effectively improve ischemic heart disease. However, the major challenge in applying hCPCs to clinical therapy is the low survival rate of graft hCPCs in the host heart, which limited the benefit of transplanted hCPCs. Bradykinin (BK) is a principal active agent of the tissue kinin-kallikrein system. Our previous studies have highlighted that BK mediated the growth and migration of CPCs by regulating Ca^2+^ influx. However, the protective effect of BK on CPCs, improvement in the survival rate of BK-pretreated hCPCs in the infarcted heart, and the related mechanism remain elusive.

**Methods:**

HCPCs were treated with H_2_O_2_ to induce cell apoptosis and autophagy, and different concentration of BK was applied to rescue the H_2_O_2_-induced injury detected by MTT assay, TUNEL staining, flow cytometry, western blotting, and mitoSOX assays. The role of autophagy in the anti-apoptotic effect of BK was chemically activated or inhibited using the autophagy inducer, rapamycin, or the inhibitor, 3-methyladenine (3-MA). To explore the protective effect of BK on hCPCs, 3-MA or BK-pretreated hCPCs were transplanted into the myocardial infarcted rats. An echocardiogram was used to determine cardiac function, H&E and Masson staining were employed to assess pathological characteristics, HLA gene expression was quantified by qRT-PCR, and immunostaining was applied to examine neovascularization using confocal microscopy.

**Results:**

The in vitro results showed that BK suppressed H_2_O_2_-induced hCPCs apoptosis and ROS production in a concentration-dependent manner by promoting pAkt and Bcl-2 expression and reducing cleaved caspase 3 and Bax expression. Moreover, BK restrained the H_2_O_2_-induced cell autophagy by decreasing LC3II/I, Beclin1, and ATG5 expression and increasing P62 expression. In the in vivo experiment, the transplanted BK- or 3-MA-treated hCPCs were found to be more effectively improved cardiac function by decreasing cardiomyocyte apoptosis, inflammatory infiltration, and myocardial fibrosis, and promoting neovascularization in the infarcted heart, compared to untreated-hCPCs or c-kit^-^ cardiomyocytes (CPC^-^ cells).

**Conclusions:**

Our present study established a new method to rescue transplanted hCPCs in the infarcted cardiac area via regulating cell apoptosis and autophagy of hCPCs by pretreatment with BK, providing a new therapeutic option for heart failure.

**Supplementary Information:**

The online version contains supplementary material available at 10.1186/s13287-021-02503-6.

## Background

Heart failure (HF) is a frequent complication of myocardial infarction (MI), found in approximately 25% of patients with MI [1]. Cardiac c-Kit^+^ progenitor cell (CPC) therapy is a potentially novel approach to treat HF caused by ischemic cardiomyopathy [2]. The falsification of data related to CPCs by a Harvard laboratory has been a veritable tragedy. However, at least 50 studies from 26 laboratories independent of the Harvard groups have reported beneficial effects of CPCs in mice, rats, pigs, and cats [3–7]. Although preclinical studies and clinical trials have demonstrated the potential of CPCs to alleviate chronic heart failure, the utmost potential of these cells has not been realized because of poor engraftment and survival. Local (intramyocardial or intracoronary injection) or systemic delivery of stem or progenitor cells may trigger loss of a considerable amount of cells (up to 90%) owing to either mechanical damage in the needle or apoptosis when the cells are administered to ischemic and inflamed tissues [8]. Thus, exploring an effective strategy that enhances CPC survival rate after adoptive transfer would have enormous therapeutic implications for ischemic heart disease.

Bradykinin (BK) is a classic endogenous vasoactive peptide that protects cells against senescence or other damage by acting as a B2 receptor [9, 10]. Our previous findings demonstrated that BK-mediated B2R activation promotes cell cycle progression and migration in hCPCs by regulating Ca^2+^ via IP3R3 and SOCE channels as well as activating PLC, PKC, pERK1/2, pAkt, and cyclin D1 signaling pathway [11, 12]. However, whether BK can protect of CPCs against H_2_O_2_-induced cell apoptosis and autophagy and whether transplantation of BK-pretreated-CPCs can improve myocardial infarction remain unknown and will be explored. 

## Methods

### Materials

BK was procured from Sigma-Aldrich (St Louis, MO, USA). Annexin V-FITC apoptosis detection Kit, 2′7′-dichlorofluorescein diacetate (DCFH-DA), α-modified Eagle’s medium (α-MEM), Iscove’s modified Dulbecco’s medium (IMDM), and fetal bovine serum (FBS) were purchased from Thermo Fisher Scientific (MA, USA). The Masson’s Staining kit was procured from Nanjing Jiancheng Bioengineering Institute (Nanjing, Jiangsu, China). MitoSOX red mitochondrial superoxide indicator was bought from Yeasen Biotech Co., Ltd. (Shanghai, China). Rat Bradykinin ELISA kit was bought from Elabscience Biotechnology (Wuhan, China). The anti-Bcl-2 (Ab59348) and anti-Bax (Ab32503) primary antibodies were purchased from Abcam Biotechnology. The anti-caspase-3 (#9662), anti-cleaved caspase 3 (#9661), anti-ATG5 (#12994S), anti-Akt (#9272S), anti-Sox2 (#3579), and anti-phosphorylation Akt (#4060S) primary antibodies were obtained from Cell Signaling Technology, and anti-Beclin-1 (PRS3616) primary antibody was from Sigma-Aldrich (St. Louis, Missouri, USA). The anti-LC3 I/II (18725-1-AP) and anti-P62 primary (18420-1-AP) primary antibodies are products of Protein Tech, whereas the anti-β-actin (SC-47778) primary antibody, anti-CD117 (SC-13508), anti-Oct-3/4 (SC-5279), and PECAM-1 Alexa fluor® 647 (SC-18916-AF647) were obtained from Santa Cruz (Dallas, TX, USA). A hematoxylin-eosin (H&E) staining kit (G1120) was bought from Solarbio Life Sciences (Beijing, China).

### Isolation of human cardiac c-Kit^+^ progenitor cells

Human cardiac c-Kit^+^ progenitor cells (hCPCs) were isolated from human atrial specimens derived from 5 patients (The demographic details of them are shown in Table 1.) who received cardiac surgery from Xiamen cardiovascular hospital, Xiamen University. The tissue collection was approved by the Ethics Committee of Xiamen cardiovascular hospital with informed consent. The cell isolation protocol is the same as described previously [11–13]. Briefly, tissues were immediately transported in oxygenated cardioplegic, washed with cold phosphate buffer saline (PBS), and minced into 1-mm pieces. The minced tissues were digested with 0.2% trypsin and 0.1% collagenase II at 37°C for 60 min with agitation. After centrifuging at 5000 rpm for 5 min, the cell pellet was cultured in a 6-well plate with α-MEM containing 10% FBS, 100 U/ml penicillin, 100 mg/ml streptomycin, 2 mM l-glutamine, 0.1 mM 2-mercaptoethanol, 5 ng/ml human basic fibroblast growth factor, and 5 ng/ml human epidermal growth factor. The c-kit-positive cells were sorted with a rabbit anti-c-kit (CD117) primary antibody (Santa Cruz Biotech, Santa Cruz, CA) and a goat anti-rabbit secondary antibody conjugated with magnetic beads (Miltenyi, Auburn, CA). The harvested cells were incubated with the anti-c-kit (CD117) primary antibody for 10 min and then incubated with magnetic beads. Cells were put into the MACS columns which were placed in the magnetic field of the MACS separator (Miltenyi) and rinsed with MACS buffer thrice to exclude un-labeled cell fraction (c-kit negative cardiomyocytes, CPC^-^ cells) and then removed from the magnetic field of the separator, and rinsed with the MACS buffer to collect the effluent solution containing magnetically retained c-kit positive cells. The collected hCPCs cells and CPC^-^ cells were seeded into flasks or plates with IMDM containing 10% FBS, 100 U/ml penicillin, 100 mg/ml streptomycin, 2 mM l-glutamine, 0.1 mM 2-mercaptoethanol, 5 ng/ml human basic fibroblast growth factor, and 5 ng/ml human epidermal growth factor. The isolated hCPCs were validated using flow cytometry with PE-connected CD8A, CD29, CD34, CD45, CD133, and CD117) and immunostaining with CD117, SOX2, and Oct-3/4 primary antibodies (Supplemental figure 1). These character of hCPCs are consistent with previous reports [4, 13, 14]. All the cells used in this study were pooled from 5 patients, and all the experiments were performed with the cells from passages 2–6.

**Table 1:** Demographic details of the human atrial sample donors

Patient number	Age	Gender	Disease	Medications
1	1 year	2	Congenital heart disease	No
2	1 year and 3 months	1	Congenital heart disease	No
3	6 months	2	Congenital heart disease	No
4	1 year and 3 months	1	Congenital heart disease	No
5	5 months	1	Congenital heart disease	No

### Cell viability assays

The 3-(4,5-dimethyl-2-thiazolyl)-2,5-diphenyl-2-H-tetrazolium bromide (MTT) assay was applied to analyze the hCPCs viability. Cells were plated onto 96-well plates in 200 μL IMDM normal cultured medium for 12 h. After pretreated with different concentrations of BK (1, 3, and 10 nM) for 1 h, cells were then induced by H_2_O_2_ (500 μM) in IMDM with 1% FBS. After 24 h incubation, the PBS-buffered MTT stock solution was applied to each well. Then, the plates were incubated for an additional 4 h. The medium was discarded, and dimethyl sulfoxide (Sigma) was added to each well. After the formazan crystals were dissolved, the optical density (OD) values were read on a microplate spectrophotometer (Bio-Tek Instruments, Winooski, VT).

### Western blotting

Western blotting was used to determine the expression of apoptosis- and autophagy-related proteins, such as pAkt, Akt, Bax, Bcl-2, caspase-3, cleaved caspase-3, LC3-I/II, Becline-1, ATG5, P62, and β-actin, with a different treatment on cultured hCPCs as described in previous studies [11–13, 15–17]. Briefly, the cells were plated in 6-well plates in 2 mL IMDM medium with 1% FBS overnight and pretreated with different concentrations of BK (1, 3, and 10 nM) or 3-methyladenin (3MA) (10 μM) for 1 h and then treated with H_2_O_2_ (500 μM) or rapamycin (10 μM) for additional 24 h. After treatment, cells were lysed by protein extraction radioimmunoprecipitation assay (RIPA) buffer (Solarbio, Beijing, China) with various protease inhibitors. Protein concentration was detected using the bicinchoninic acid (BCA) protein assay Kit (Solarbio, Beijing, China). The protein samples were mixed and denatured with loading buffer and separated via 10% or 12% polyacrylamide gel electrophoresis and transferred onto polyvinylidene fluoride membranes. After that, membranes were blocked with nonfat milk in Tris-buffered saline Tween-20 (TBST) buffer and then incubated with corresponding primary antibodies. After incubating with Horseradish Peroxidase (HRP)-conjugated secondary antibodies for 1 h, the membranes were washed and exposed using enhanced chemiluminescence (ECL) with a detection system (FluoChem E, CA, USA). The target proteins band were quantitatively analyzed using the ImageJ analysis software (NIH Image).

### Flow cytometry analysis

The cell apoptosis and intracellular reactive oxygen species (ROS) production in hCPCs were determined using a flow cytometer (Cytoflex, Beckman Coulter, USA). For apoptotic analysis, after pretreating with different concentrations of BK (1, 3, and 10 nM) or 3-methyladenine (3-MA; 10 μM) for 1 h, the hCPCs were treated with H_2_O_2_ (500 μM) or rapamycin (10 μM) for additional 24 h. Then, the cells were collected and stained using the Annexin V-FITC Apoptosis Detection Kit (Dojindo Molecular Technologies, Japan) according to the manufacturer’s instructions.

For ROS production determination, after different treatments, cells were collected and incubated with DCFH-DA (10 μM) at 37°C for 30 min, then the intracellular ROS level was determined using a flow cytometer (Cytoflex, Beckman Coulter, USA).

### TUNEL assays

A kit of terminal deoxynucleotidyl transferase (TdT)-mediated dUTP nick end labeling (TUNEL) from Roche was used to determine cell apoptosis in vivo and in vitro. The procedures were performed according to the manufacturer’s protocol (Roche, Germany). Briefly, after treatment with BK or 3MA for 1 h, the hCPCs were exposed to H_2_O_2_ or rapamycin for 24 h and then washed with DNase-free water. Cells or slides were then incubated in TdT which was labeled with FITC-conjugated secondary antibody and stained with DAPI for nucleus staining. The percentage of apoptotic cells was calculated as the ratio of TUNEL-positive cells to the total nuclei which were analyzed with the ImageJ software (NIH Image).

### Mitochondrial superoxide assay

Approximately, 1x 10^5^ hCPCs/ml were seeded onto 6 well plates. After treatment with different concentrations of BK or/and H_2_O_2_, cells were incubated with a 5-μM MitoSOX reagent (Yeasen, Shanghai Chain). After 10 min of incubation at 37°C in the dark, the plates were washed three times using HBBS washing buffer and fixed with 4% PFA. The images were obtained using a fluorescence microscope (Thermo Fisher Scientific, USA). The fluorescence intensity of images was quantified using the ImageJ analysis software (NIH Image).

### Animal experiments

The healthy male Sprague–Dawley (SD) rats (aged 8–10 weeks, weighing 250–300g, purchased from the Shanghai SLAC Laboratory Animal Co., Ltd., Shanghai, China) were bought for our experiments. All experiments complied with the guiding principles of laboratory animals in Xiamen University and were approved by the Committee for Animal Experimentation. For the rat myocardial infarction (MI) model, the rats were anesthetized and the left anterior descending coronary artery was ligated. The limb leads of ECG recordings were employed to determine the success of MI surgery with ST-segment elevated. The sham-operated rats were subjected to the same surgery except for the snare, which was left untied. Preconditioned-hCPCs were collected and resuspended in PBS for the following transplantation after treatment with BK or 3-MA for 24 h. After 15 min of ischemia, the heart was injected with 1×10^6^ hCPCs based on previous literature showing therapeutic benefit [18]. The injected cells or vehicle (PBS) was delivered in the free wall of the left ventricle (LV) under direct vision with 5 points which mirrored the region of the heart most affected by MI. Six weeks after CPC-transplantation, the echocardiographic assessment was conducted to estimate left ventricular function. After that, all the rats were euthanized by blood withdrawal in deep anesthesia with isoflurane, and the heart tissues and blood were collected.

### Echocardiographic assessment

Six weeks after the MI surgery, an echocardiographic assessment (Vevo 2100 Imaging System of Visual Sonics Inc.) was conducted to assess the rat cardiac structure and function. The echocardiographic parameters including left ventricular ejection fraction (LVEF), left ventricular shortening fraction (LVFS), left ventricular end-diastolic diameter (LVEDd), and left ventricular end-systolic diameter (LVESd) were determined by M-mode and LV long-axis view. Subsequently, the rats were euthanized and the hearts were rapidly excised, weighed, and used for histological examination.

### Hematoxylin-eosin (H&E) staining

After echocardiographic data assessed, rats were euthanized by blood withdrawal in deep anesthesia with isoflurane. The myocardial tissues were fixed in a tissue freezing medium with optimal cutting temperature compound, then frozen in liquid nitrogen for a few seconds, stored in −80° refrigerator, and sectioned 6-μm thick for the experiment. H&E staining was used to assess cardiac morphology. The sections were fixed in 4% paraformaldehyde, washed with ddH_2_O, stained with hematoxylin, differentiated in 1% HCl-ethanol, washed thoroughly, soaked in ammonia water till the nucleus color became blue, and stained in eosin. Then, the sections were dehydrated by gradient alcohol, made transparent in xylene twice, and then sealed with neutral resin. Finally, the tissues were dried for 48–72 h and then photographed. The morphometric parameters in five slices of each heart including total LV area and scar area were blindly analyzed, and the scar size was calculated as the total scar area divided by the LV area.

### Masson’s staining

Masson’s trichrome staining was applied to determine myocardial fibrosis. The heart samples were sectioned and fixed. The sections were subjected to Masson’s staining based on the instructions of the Masson’s Staining Kit (Nanjing Jiancheng Bioengineering Institute, Nanjing, China). The fibrotic size was determined as the average ratio of the fibrotic area to the LV area using the ImageJ analysis software (NIH Image).

### Quantitative real-time PCR analysis for HLA-A gene expression

The total RNA was isolated from the rat heart tissue using the TRIZOL reagent (Thermo Fisher). A total RNA of 1 μg was used to reverse-transcribe to cDNA with the First-Strand cDNA Synthesis Kit (TAKARA Biomedical Technology), and Quantitative Reverse Transcription PCR was performed using the SYBR Green PCR Kits (TAKARA Biomedical Technology) on Bio-Rad CFX96 Real-Time System. The relative HLA-A gene expression was normalized to GAPDH. The primer sequences are as follows: human *HLA-A* (F: 5′-ATCATTGCTGGCCTGGTTCTC-3′; R: 5′-CACACAAGGCAGCTGTCTCAC-3′), *GAPDH* (F: 5′-TGACAACTCCCTCAAGATTGTCA-3′; R: 5′-GGCATGGACTGTGGTCATGA-3′).

### Immunofluorescence staining

The heart sections were fixed with acetone for 15 min on ice, then blocked in normal goat serum for 60 min after permeabilization with 0.3% Triton X-100 in PBS for 10 min, and then sections were incubated with PECAM-1 (CD31) antibody (MEC 13.3) (1:200) which was coupled with Alexa Fluor® 647 fluorescence secondary antibody (Santacruz, San Francisco, USA) for 1 h. Finally, DAPI (Solarbio, Beijing, China) was used to stain the nucleus. For proliferated hCPC detection, the human-specific rabbit c-kit antibody and the proliferation-related protein Ki67 staining were applied. Briefly, after fixation with acetone for 15 min, the sections were blocked and permeabilized with 0.3% Triton X-100. The c-kit primary antibody (SC-13508) and Ki67 primary antibody (ab16667) were used to incubate the section overnight at 4°C. After washing with PBS, the sections were incubated with Alexa Fluor® 488- or PE-conjunctsecondary antibodies for 1 h and DAPI was used for staining the nucleus. The fluorescence intensity of images was quantified using an ImageJ software.

### Statistical analysis

All the data were analyzed and organized using GraphPad Prism 8.0 (GraphPad Software, Inc., San Diego, CA, USA). The results of at least three independent experiments are presented as mean ± SEM. One-way ANOVA followed by the Tukey-test was used for comparisons of multiple groups. A value of *P* < 0.05 was considered statistically significant.

## Results

### Effects of BK on H_2_O_2_-induced hCPC death and apoptosis

**Fig. 1:**
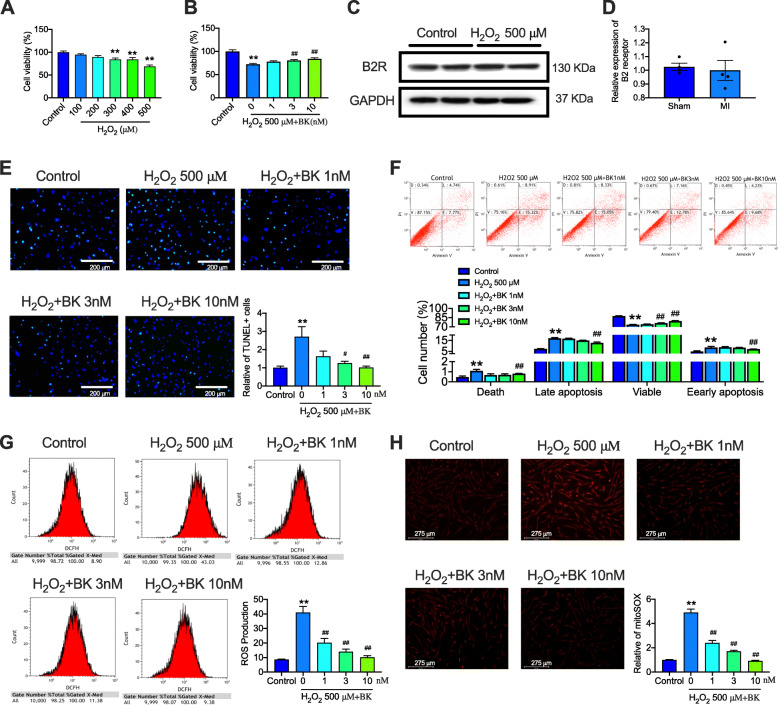
BK alleviated H_2_O_2_-induced cell apoptosis and ROS production in hCPCs. **A** Relative viability of hCPCs in the presence of different concentrations of H_2_O_2_ (100, 200, 300, 400, and 500 μM) detected with MTT. **B** Relative viability of the H_2_O_2_-induced hCPCs upon treatment with BK (1, 3, 10 nM) detected with MTT. **C–D** Representative image and quantitative analysis of B2 receptor in H_2_O_2_-induced hCPCs detected by western blot assay. **E** Representative image and quantitative analysis of TUNEL staining in hCPCs induced by H_2_O_2_ and treatment with 1, 3, 10 nM BK (Green staining cells indicated TUNEL-positive cells). **F** Representative image and quantitative analysis of in hCPCs induced by H_2_O_2_ and treatment with BK (1, 3, 10 nM) detected by Annexin V assay with flow cytometry. **G** Representative of image and quantitative analysis of flow cytometry detected the ROS production in hCPCs induced by H_2_O_2_ and treatment with BK (1, 3, 10 nM). **H** Representative of image and quantitative analysis of mitoSOX in hCPCs induced by H_2_O_2_ and treatment with BK (1, 3, 10 nM). (Data are shown as mean ± SEM, *n* = 5, *P < 0.05, **P < 0.01 VS. Control; #*P* < 0.05, ##*P* < 0.01 VS. H_2_O_2_.)

We first explored whether BK had a protective effect on H_2_O_2_-induced hCPC death. A high amount of hCPC death was induced by H_2_O_2_ in a concentration-dependent manner, especially at 500 μM, and the cell viability decreased to approximately 70% (*P* < 0.01 vs. control) (Fig. 1A). We, therefore, utilized 500 μM H_2_O_2_ in the subsequent experiments to investigate whether BK could protect hCPCs from H_2_O_2_-decreased cell viability. After pretreating with different concentrations of BK (1, 3, and 10 nM), the percentage of cell viability significantly increased in a concentration-dependent manner (*P* < 0.01 vs. H_2_O_2_-treated hCPCs) (Fig. 1B). In our previous study, we found that BK exerts its effect via the B2 receptor (B2R); hence, we also observed the expression of B2R under H_2_O_2_ stress. The results showed that treatment with H_2_O_2_ did not affect the expression of B2R in hCPCs (Fig. 1C, D). These results indicated that BK ameliorated H_2_O_2_-decreased hCPC viability and B2R was not involved in this process.

TUNEL staining and flow cytometry with Annexin V-FITC Apoptosis Detection Kit were utilized to further explore the protective effect of BK on H_2_O_2_-induced apoptosis in hCPCs. As shown in Fig. 1E, BK at 3 and 10 nM significantly decreased the proportion of TUNEL-positive cells induced by H_2_O_2_. In addition, flow cytometry detection showed that the number of viable cells was significantly decreased and late and early apoptotic cells were increased in H_2_O_2_-treated hCPCs; however, when pre-treated with BK, these effects of H_2_O_2_ were reversed (Fig. 1F).

It is widely recognized that ROS plays a crucial role in mediating cell viability and cell apoptosis especially after exposure to H_2_O_2_. To further explore whether the protective effect of BK is related to the inhibition of intracellular ROS production, we examined the ROS level in hCPCs induced by H_2_O_2_ in the presence or absence of BK by flow cytometry and mitochondrial superoxide assay. As shown in Fig. 1G and H, ROS production and mitochondrial superoxide level were greatly increased in the H_2_O_2_-treated hCPCs. When pre-treated with BK, this increase in ROS level was significantly decreased by BK. These results demonstrate that BK effectively promoted cell viability and decreased late and early apoptosis as well as the cellular ROS level induced by H_2_O_2_ in hCPCs.

### Signal molecules involved in the anti-apoptosis effect of BK in hCPCs

**Fig. 2:**
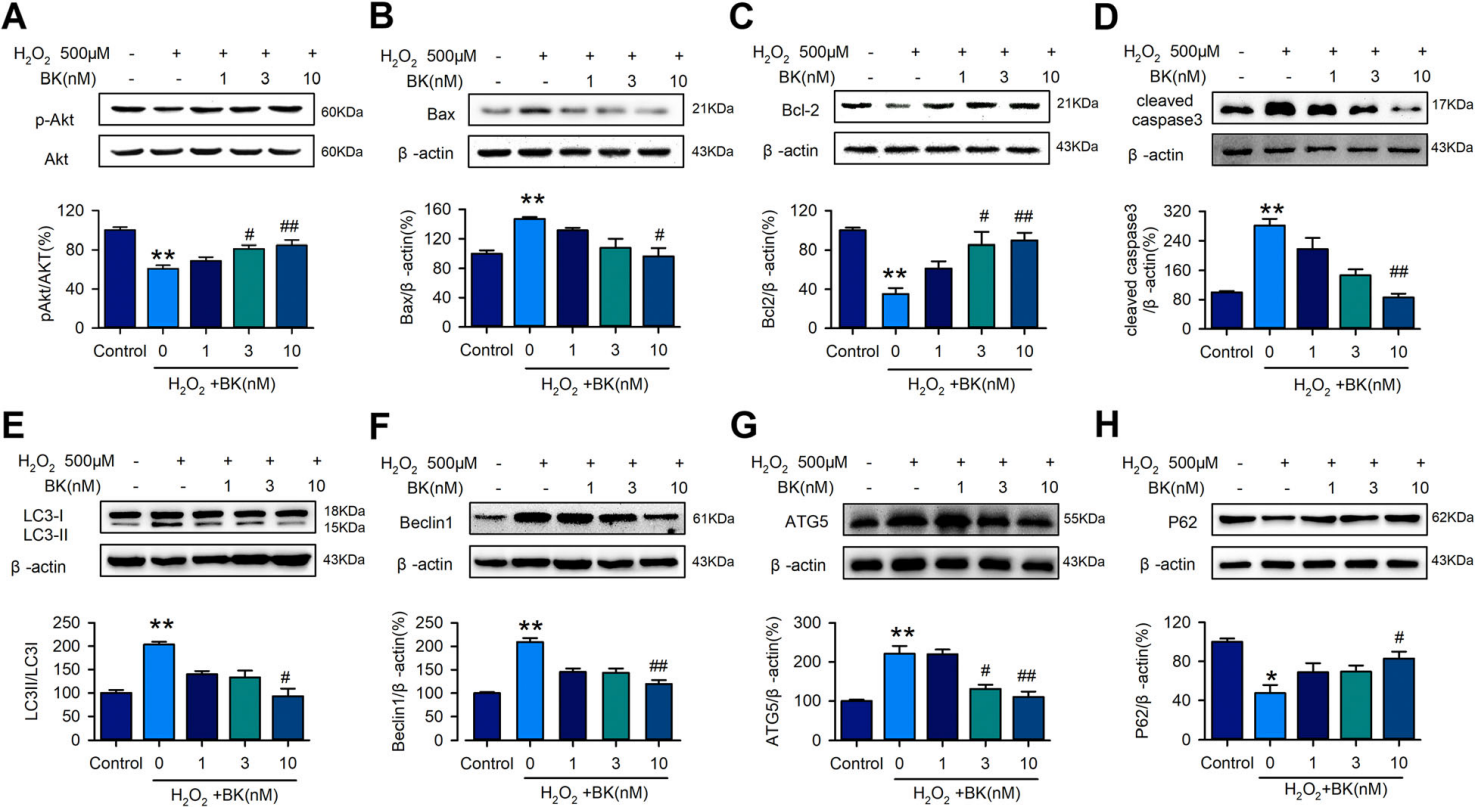
BK regulated the H_2_O_2_-induced apoptosis- and autophagy-related proteins. Representative of image and quantitative analysis of western blot assay detected apoptosis-related proteins, pAkt (**A**), Bax (**B**), Bcl-2 (**C**), and cleaved-caspase 3 (**D**) in hCPCs induced by H_2_O_2_ and treatment with BK (1, 3, 10 nM). Representative of image and quantitative analysis of western blot assay detected autophagy-related proteins, LC3II/I (**E**), Beclin 1 (**F**), ATG5 (**G**), and P62 (**H**) in hCPCs induced by H_2_O_2_ and treatment with BK (1, 3, 10 nM). (Data are shown as mean ± SEM, *n* = 5, *P < 0.05, **P < 0.01 VS. Control; #*P* < 0.05, ##*P* < 0.01 VS. H_2_O_2_)

The molecular proteins related to apoptosis were determined by western blotting. The results showed that exposure to H_2_O_2_ downregulated phosphorylation of Akt (Fig. 2A) and Bcl-2 (Fig. 2C), and upregulated Bax (Fig. 2B) and cleaved caspase 3 (Fig. 2D), whereas BK reversed the effect of H_2_O_2_ in a concentration-dependent manner, especially at 10 nM. These results illustrated that BK regulates cell apoptosis by upregulating anti-apoptosis proteins Bcl-2 and pAkt and downregulating pro-apoptosis proteins cleaved caspase-3 and Bax.

Autophagy is an important regulator in the progress of cell apoptosis. We also analyzed the signal molecular proteins related to autophagy involved in BK-regulated cell apoptosis. The results of western blotting showed that compared to the control group, LC3-II/I (Fig. 2E), Beclin1 (Fig. 2F), and ATG5 (Fig. 2G) levels in the H_2_O_2_-treated hCPCs were significantly increased and P62 (Fig. 2H) expression was dramatically decreased. In contrast to the H_2_O_2_-treated cells, BK reversed H_2_O_2_-induced autophagy in hCPCs in a concentration-dependent manner. These results demonstrate that cell autophagy may involve BK-regulated hCPC apoptosis via regulating autophagy-related proteins.

### Autophagy was involved in the role of BK in hCPC apoptosis

**Fig. 3:**
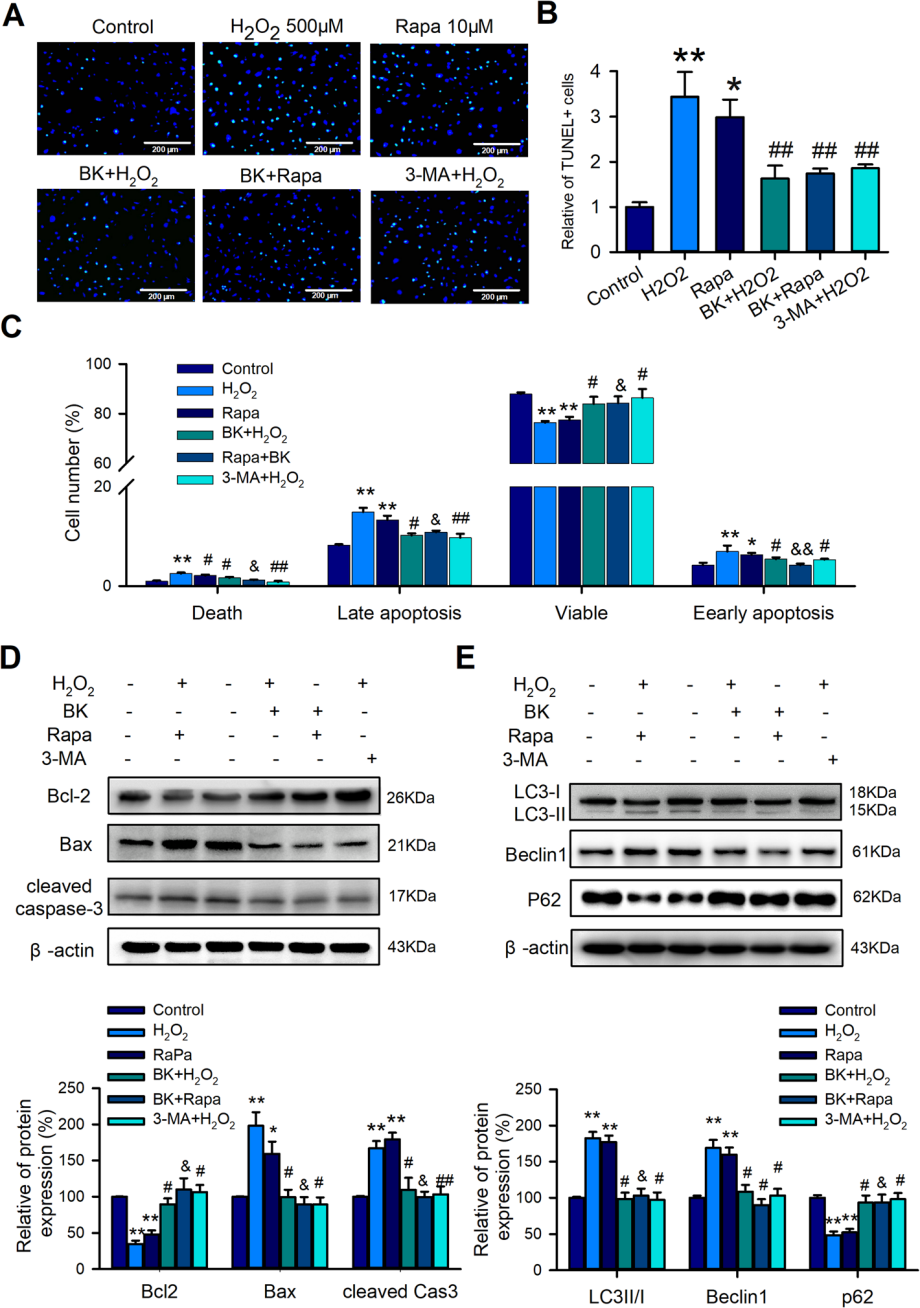
Autophagy participated in the BK-regulated apoptosis induced by H_2_O_2_ in hCPCs. **A–B** Representative image and quantitative analysis of TUNEL staining in hCPCs induced by H_2_O_2_ or rapamycin and treatment with BK or 3-MA. **C** Quantitative analysis of Annexin V flow cytometry assay in hCPCs induced by H_2_O_2_ or rapamycin and treatment with BK or 3-MA. **D** Apoptosis-related protein expression, including Bax, Bcl-2, cleaved caspase 3, induced with H_2_O_2_ or rapamycin, and treatment with BK or 3-MA. **E** Autophagy-related protein expression, including LC3II/I, Beclin 1, and P62, induced with H_2_O_2_ or rapamycin and treatment with BK or 3-MA. (Data are shown as mean ± SEM, *n* = 5, *P < 0.05, **P < 0.01 VS. control; #*P* < 0.05, ##*P* < 0.01 VS. H_2_O_2;_ &*P* < 0.05, &&*P* < 0.01 VS. Rapa)

To further validate whether autophagy is involved in BK-regulated cell apoptosis, autophagy activator rapamycin (Rapa) and inhibitor 3-MA were introduced in the subsequent experiments. Interestingly, similar to the effect of BK, the H_2_O_2_- or Rapa-induced TUNEL-positive cells as well as the late and early apoptosis cells can be dramatically reversed by 3-MA, and BK also decreased the autophagy activator Rapa-induced cell apoptosis (Fig. 3A, B, and C). We further explored signal molecules involved in BK-regulated autophagy and the effect of the activator and inhibitor of autophagy on the molecules of cell apoptosis and autophagy. Similar to the results of the TUNEL assay and Annexin V Flow cytometry detection, the expression levels of pro-apoptosis protein Bax and cleaved Caspase3 were decreased and the level of anti-apoptosis protein Bcl-2 was increased by BK or 3-MA in H_2_O_2_- or Rapa-induced apoptosis (Fig. 3D). In addition, H_2_O_2_- or Rapa-induced expression alteration of LC3 II/I, Beclin1, and P62 can be significantly reversed by BK or 3-MA (Fig. 3E). These results indicated that autophagy is involved in BK-regulated apoptosis in hCPCs.

### BK-treated hCPCs significantly improved the cardiac function of MI rats

**Fig. 4:**
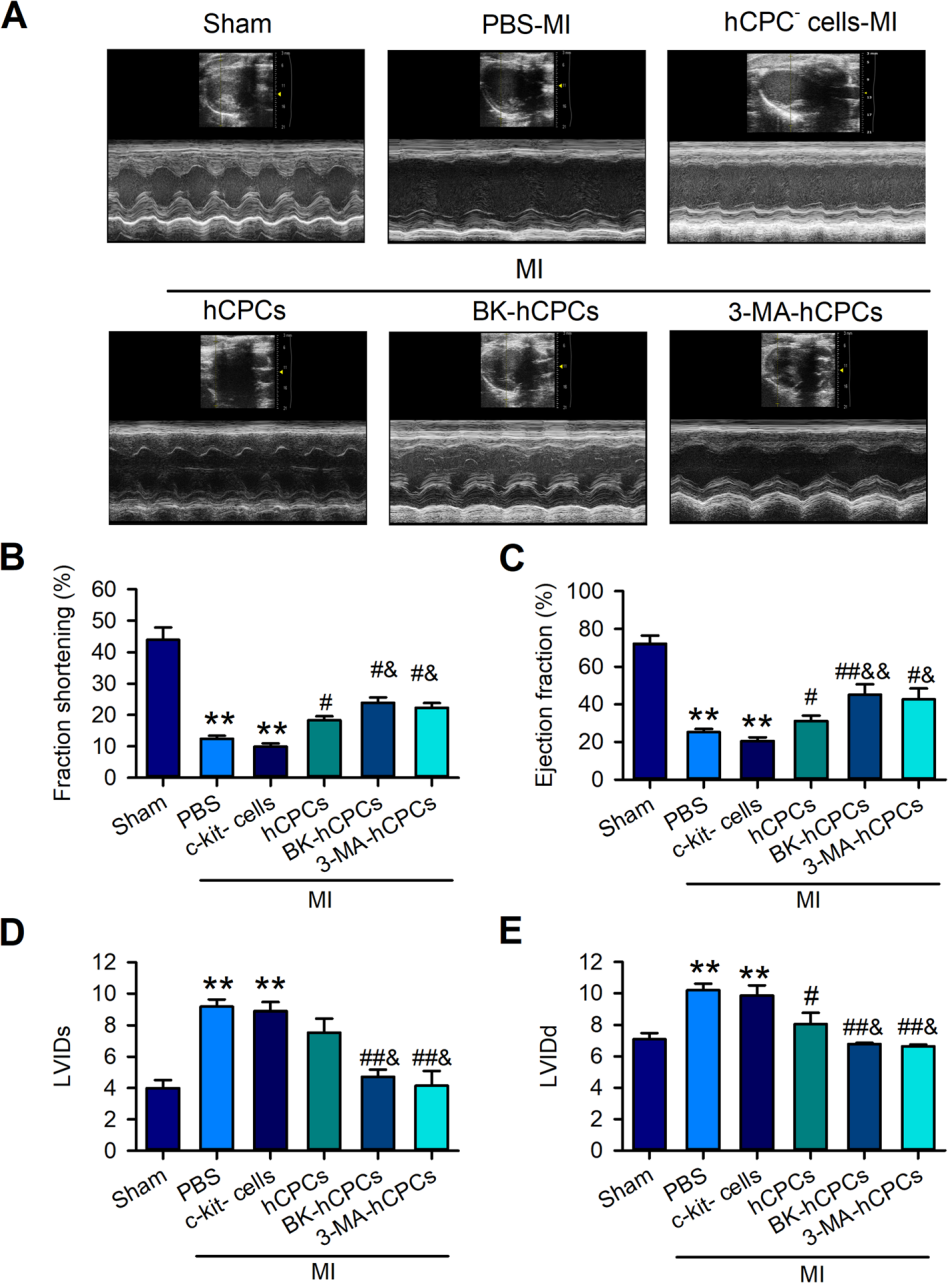
BK-treated hCPCs improved left ventricular function in MI rats. **A** Representative image of the echocardiography detected at 6 weeks after MI. Data analysis of the left ventricular fractional shorting (LVFS) (**B**), ejection fraction (LVEF) (**C**), left ventricular internal diameter at end-systole (LVIDs) (**D**), and left ventricular internal diameter at end-diastole (LVIDd) (**E**) were showed in the sham group (Sham), MI rats treated with the PBS group (PBS), MI rats transplanted with the hCPC-negative cell group (hCPC^-^ cells), MI rats transplanted with the hCPC group (hCPCs), MI rats transplanted with the BK-treated hCPC group (BK-hCPCs), and MI rats transplanted with the 3-MA-treated hCPC group (3-MA-hCPCs). (Data are shown as mean ± SEM, *n* = 6, **P < 0.01 VS. Sham; #*P* < 0.05, ##*P* < 0.01 VS. MI; &*P* < 0.05 VS. hCPCs-MI)

To examine the in vivo protective effect of BK on hCPCs in MI, we transplanted the BK-pre-treated CPCs into MI rats and explored the effect of BK-pre-treated hCPCs on MI cardiac function, inflammation, and cardiac fibrosis; c-kit-negative cardiomyocytes (CPC^-^ cells) and PBS-treated-hCPCs were employed as the control cells. The echocardiographic function including LVFS, LVEF, LVIDs, and LVIDd in rats transplanted with different treatments of hCPCs was detected before the rats were euthanized. In contrast to the CPC^-^ cells-transplanted rats, the levels of LVFS (Fig. 4B), LVEF (Fig. 4C), LVIDs (Fig. 4D), and LVIDd (Fig. 4E) were slightly improved in the PBS-treated-hCPC transplanted group (CPCs). Interestingly, these indicators of LV function were significantly improved by the BK or 3-MA-treated hCPCs.

**Fig. 5:**
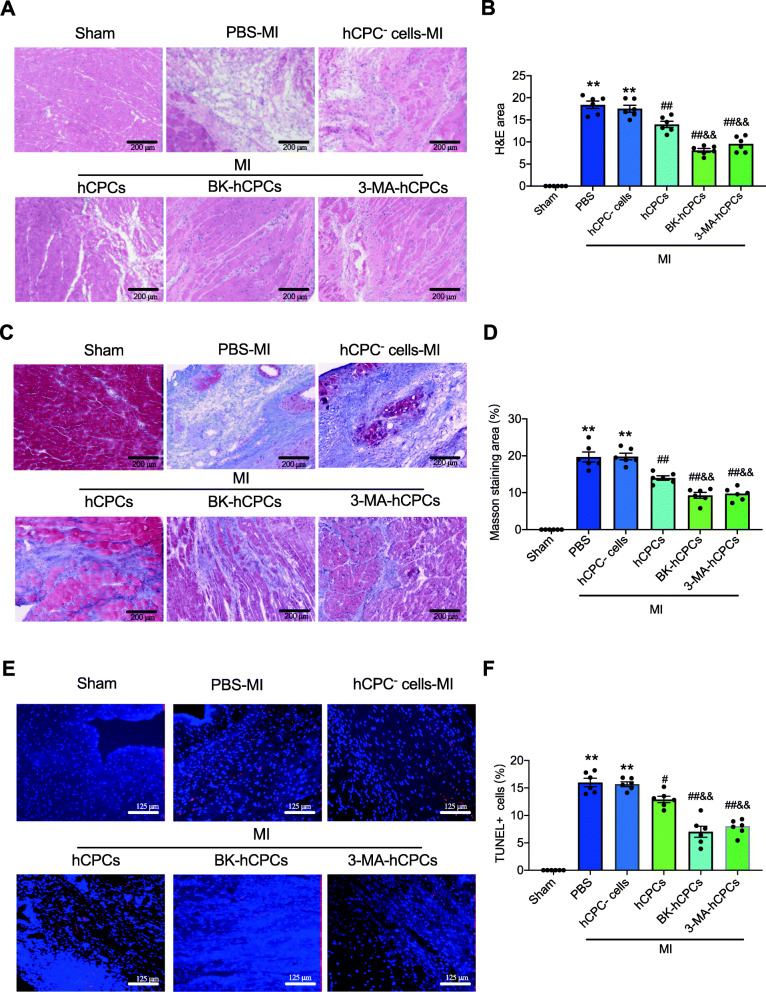
BK-preconditioned hCPCs reduced myocardial inflammation, fibrosis, and cell apoptosis in MI rats. Representative images (**A**) and quantitative analysis (**B**) of H&E staining 6 weeks after MI in the sham group (Sham), MI rats treated with the PBS group (PBS), MI rats transplanted with the hCPC-negative cell group (hCPC^-^ cells), MI rats transplanted with the hCPC group (hCPCs), MI rats transplanted with the BK-treated hCPC group (BK-hCPCs), and MI rats transplanted with the 3-MA-treated hCPC group (3-MA-hCPCs). Representative images (**C**) and quantitative analysis (**D**) of Masson’s staining 6 weeks after MI. Representative protein expression (**E**) and quantitative analysis (**F**) of TUNEL staining in corresponding groups. (Data are shown as mean ± SEM, *n* = 6, **P < 0.01 VS. Sham; #*P* < 0.05, ##*P* < 0.01 VS. MI; &*P* < 0.05, &&*P* < 0.01 VS. CPCs-MI.)

A similarly protective effect of BK-treated hCPCs was observed in pathological results, such as H&E staining, Masson’s staining, and TUNEL staining. As shown in Fig. 5, transplantation with BK- or 3-MA-treated CPCs in MI rats showed significantly decreased inflammatory cell infiltration (Fig. 5A and B), cardiac fibrosis (Fig. 5C and D), and cardiomyocyte apoptosis (Fig. 5E and F) in the infarcted area, compared to CPC^-^ cells or hCPC-transplanted MI rats. These results suggested that BK-pretreated hCPCs have a more protective effect than CPC^-^ cells or hCPCs on cardiac function in myocardial infarction rats by restrained inflammatory cell infiltration, myocardial fibrosis, and cardiomyocyte apoptosis.

### Pretreatment with BK on hCPCs significantly promoted the survival rate of hCPCs in MI heart

**Fig. 6:**
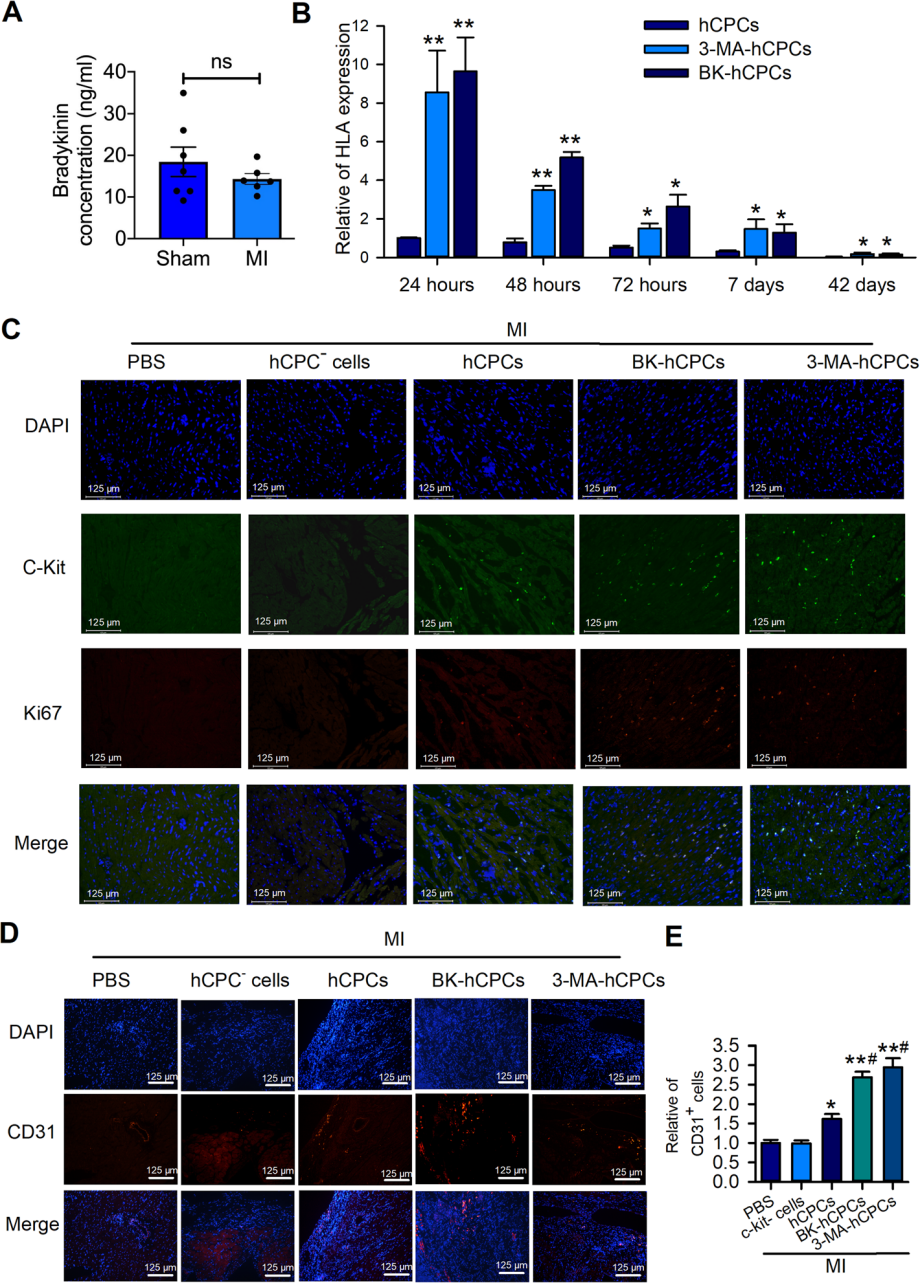
BK elevated hCPCs survival rate and revascularization in MI heart. **A** ELISA assay showed the concentration of bradykinin in sham and MI rats. **B** RT-qPCR detection showed the human-specific gene expression in rat hearts at 24 h, 48 h, 72 h, 7 days, and 42 days after transplantation in different groups. **C** Representative of fluorescence images of c-kit and Ki67 in the border area of MI rat hearts after transplanted 24 h. Representative of fluorescence (**D**) images and quantitative analysis (**E**) of CD31 in the border area of MI hearts 42 days after transplanted with different cells. (Data are shown as mean ± SEM, *n* = 6, *P < 0.05, **P < 0.01 VS. MI; #*P* < 0.05, ##*P* < 0.01 VS. CPCs-MI)

To determine the role of BK in myocardial infarction, we first detected the concentration of BK in serum at the baseline and 24 h after inducing MI in rats. The results showed that the serum concentration of BK in the MI group was decreased slightly but without a significant difference (Fig. 6A), which indicated that BK may play an important role in MI-induced injury. Previous studies have reported that grafted CPCs showed a low survival rate in the first few days after transplantation in MI hearts. To determine the survival rate of transplanted BK-treated hCPCs in the MI heart, we determined the expression of human-specific gene HLA-A after transplantation at 24 h, 48 h, 72 h, 7 days, and 42 days. The expression of HLA-A in the heart which was transplanted with BK- or 3-MA-treated hCPCs showed a higher expression compared to that in rats transplanted with untreated-hCPCs after transplantation at 24 h, 48 h, 72 h, 7 days, and 42 days (Fig. 6B).

To examine the mechanism involved in the protection of BK-treated hCPCs in MI rats, we determined the proliferated hCPCs in the border area of the infarcted heart using a human-specific c-kit primary antibody and a marker of proliferation-related protein Ki67 24 h after transplantation. As shown in Fig. 6C, we did not find c-kit- or Ki67-positive cells in PBS or c-kit-negative cells (CPC^-^ cells) transplanted hearts. However, a few c-kit-positive cells were observed in hCPC-transplanted rats and exhibited a slightly proliferative character. Interestingly, the BK- or 3-MA-treated hCPC group showed more c-kit-positive cells and Ki67-positive cells compare to other groups. These results indicated that BK-treated hCPCs exhibit a higher survival rate than that of un-treated hCPCs in MI rats by inhibiting cell autophagy.

Several previous studies demonstrated that CPCs have an angiogenic effect on MI, but whether BK-treated hCPCs exhibit a higher angiogenic effect than untreated hCPCs has not yet been explored. Interestingly, although the transplanted cells exhibit a small amount 42 days after grafting, neovascularization was much more prominent in rats transplanted with BK- or 3-MA-treated cells than in those transplanted with untreated-hCPCs (Fig. 6D and E). These results indicated that although BK-preconditioned hCPCs exhibited a low survival rate after transplantation for a long time in MI rats, owing to their paracrine role, hCPCs may exert a sustained effect on revascularization in the ischemic heart.

## Discussion

Cardiac stem/progenitor cells are a heterogeneous group of cells distributed throughout different regions of the heart, including the ventricle, atria, pericardium, or epicardium [19]. Among the various cardiac stem/progenitor cells, cardiac c-Kit^+^ progenitor cells (CPCs) play an important role and offer greater growth potential than other cardiac progenitor cells [20]. For the past decade, CPC therapy has been regarded as a promising approach for ischemic heart disease [14]. Clinical trials reported that human CPC transplantation in patients with acute MI injury or heart failure have expanded worldwide. However, one of the major challenges associated with stem/progenitor cell therapy is the new consensus that directly transplanted stem/progenitor cells do not survive too long in the host ischemic heart [5]. Therefore, finding a new approach to prolong the survival rate of CPCs to improve the host cardiac function is an important challenge for researchers. Various approaches have been used to overcome this problem, including preconditioning stem cells by genetic or pharmacologic manipulations or exposing them to in vitro conditions that mimic the harsh environment of an ischemic heart, such as preconditioning with cobalt protoporphyrin in CSCs [21], cobalt chloride in human ESCs [22], pioglitazone in MSCs [23], argon in human cardiac myocyte-like progenitor cells [24], overexpression of Pim-1 in hCPCs [25], and pretreatment of CSCs with MSC exosome [26]. Although all of these approaches showed a potential role in improving cardiac function, the quantitative comparison of transplanted cells in the host heart and their anti-apoptosis and pro-angiogenesis effect has not been demonstrated.

BK is a major kinin substance of the Kallikrein-Kinin System (KKS) that acts in conjunction with the target cells. The BK receptors B1Rs and B2Rs, which are G-protein-coupled receptors, are involved in a variety of systems and organs [27, 28]. Accumulation of evidence corroborates that the protective effect of KKS on the ischemic heart was mainly achieved by B2 receptors [29, 30]. Experimental MI in rats induces complex degradation in the metabolism of exogenous BK, which results in an increased relative contribution of neutral endopeptidase after infarction [31]. This finding is consistent with our results that the concentration of BK slightly decreased in MI rats after 24 h. Another study reported that BK reduced the extent of MI via B2 receptor signaling because of the increase in regional myocardial blood flow around the ischemic lesion [32], which indicated that preconditioning with BK may have the beneficial potential to protect stem/progenitor cells. Interestingly, several independent research groups reported the potential protective role of BK in stem/progenitor cells, including endothelial progenitor cells (EPCs), neural progenitor cells (NPCs), and CPCs. In vitro, BK was applied to protect CPCs from high-glucose-induced senescence [10], and EPCs from oxidative stress-induced senescence [33], as well as enhance neuron-generating division of NPCs [34]. In addition, two other studies determined the protective effect of BK on EPCs in vivo; one of them showed that BK-treated EPCs showed robust cell anti-apoptosis, reduction in infarct size, and significant improvement in cardiac function [35], and the other one showed that BK preconditioning increased EPC migration, tube formation, and neovascularization and improved cardiac function following transplantation [36].

Our previous studies have showed that hCPCs expressed B2 receptors but not B1 receptors, and treatment of hCPCs with BK promoted cell proliferation and migration by regulating Ca^2+^ influx via the IP3 receptor and SOCE channel as well as the PLC/Akt/ERK pathway [11, 12]. However, whether BK exerts an anti-apoptotic effect in vitro and whether transplantation of BK-preconditioned hCPCs in infarcted myocardium results in a significant increase in hCPC numbers and improves the left ventricular (LV) functional in vivo remain unclear. This study was undertaken to answer these questions. In the present study, in vitro, we found that BK exerts a formidable anti-apoptotic effect on cultured CPCs by inhibiting pro-apoptosis protein Bax and cleaved caspase 3 and increases the expression of anti-apoptosis protein, Bcl-2. These protective effects of BK in vitro are consistent with the findings of the abovementioned in vitro studies on the effect of BK on high-glucose-induced senescence of CPCs [10], oxidative stress-induced senescence of EPCs [33], and NPCs [34].

Autophagy has emerged as an important intracellular signaling pathway for the regulation of cellular activities including cell apoptosis [37]. Accumulated evidence showed that dysfunction of autophagy may be closely related to an imbalance in cellular metabolism, including mitochondrial dysfunction, ROS overload, and cell apoptosis [38]. In the present study, the results demonstrated that H_2_O_2_ significantly induced dysfunction of autophagy in hCPCs by increasing the autophagosome formation markers LC3II/I and Beclin-1 expression and decreasing P62. Interestingly, the H_2_O_2_-induced autophagy dysfunction of hCPCs can be reversed and protected by BK. The results of the pro-autophagy effect of H_2_O_2_ in CPCs are consistent with previous evidences, such as H_2_O_2_-induced autophagy dysfunction in c-Kit^+^ CPCs can be reversed by miR-143 via targeting Atg7 [39], and H_2_O_2_ induced autophagy dysfunction in cardiac Sca-1^+^ cells [40]. Moreover, we corroborated the viewpoint that the protective role of BK in apoptosis is regulated by targeting autophagy using an autophagy inhibitor 3-MA and inducer rapamycin. The results showed that H_2_O_2_-induced cell apoptosis and autophagy can be restrained by 3-MA, while the rapamycin-induced cell apoptosis and autophagy have been reversed by BK. These results confirmed the concept that the anti-apoptosis effect of BK on hCPCs is linked to the BK-regulated autophagosome formation. The concept that inhibition of excessive autophagy in hCPCs can attenuate damage of progenitor cells and suppression of excessive autophagy can improve myocardial injury in our present study is consistent with many other results, such as rapamycin treatment of endothelial progenitor cells (EPC) induces apoptosis and autophagy and 3-MA inhibits the proliferation and promotes apoptosis of EPC [41], monotropein promotes angiogenesis and inhibits oxidative stress-induced autophagy in endothelial progenitor cells [42], hesperidin reduces myocardial I/R injury by suppressing excessive autophagy via PI3K/Akt/mTOR pathway [43], and inhibition of autophagy results in improved protection of ischemia precondition against diabetic myocardial ischemia/reperfusion injury [44].

The most extensive characteristics of myocardial infarction are excessive oxidative stress, apoptosis, and over autophagy of cardiomyocytes. Previous reports have demonstrated that after graft of progenitor/stem cell to the infarcted myocardium, almost all of the cells were dead after transplanted in the ischemia circumstance with excessive oxidative stress and excessive inflammatory filtration [3, 19, 45]. However, little is known about whether the anti-apoptotic effect of BK on CPCs can improve cardiac function in MI rats. In our present study, the protective effect and the regulation mechanism of BK on CPCs in vivo were explored by intra-cardiac injection with BK-treated CPCs in the MI rats. The results showed that LVEF, LVFS, LVIDd, and LVIDs were slightly increased in hCPC-transplanted rats (CPCs), whereas these echocardiographic parameters showed significant improvement in the BK-treated CPC group or 3-MA-treated CPC group. Our results also corroborate that BK-treated hCPCs contribute to exert anti-inflammatory infiltration, anti-cardiac fibrosis, and inhibiting cardiomyocyte apoptosis in the infarct area of the MI rat heart, which may be relevant to the role of BK in regulating cell autophagy.

There are still many limitations to the present study. First, further mechanisms of BK-regulated autophagy of hCPCs were not explored. Second, although we have observed transplanted-hCPCs promoted angiogenic effect in MI rats after 42 days and speculated that the paracrine role of hCPCs may play a key role in revascularization in hCPC-transplanted MI heart, the deep mechanisms of the BK-promoted paracrine effect on hCPCs were not investigated in this study. Lastly, the angiogenic effect of the BK-treated hCPCs should be further verified through in vitro and in vivo experiments, such as tube formation detection in endothelial cells and in hind-limb ischemic mice.

## Conclusions

Collectively, the findings of the present study can be summarized as follows: (a) BK attenuates H_2_O_2_-induced CPCs apoptosis and ROS production in a concentration-dependent manner, which promoted the levels of pAkt and Bcl-2 as well as reduced cleaved caspase 3 and Bax; (b) The anti-apoptotic effect of BK is related to the regulation of cell autophagy by decreasing LC3II/I, Beclin1, and ATG5 levels, which was confirmed using the autophagy inhibitor 3-MA and autophagy activator rapamycin; (c) We also found that grafting with the BK-treated CPCs or 3-MA-treated CPCs can significantly improve cardiac function by decreasing cardiomyocyte apoptosis, inflammation infiltration, and myocardial fibrosis in myocardial infarction rats; (d) Most interestingly, BK-preconditioning elevated the survival rate of hCPCs after transplantation, and although small quantities of CPCs have been found after 42 days of transplantation, the revascularization in BK-treated CPC-grafted rats was significantly increased, which indicated the paracrine role of BK-treated CPCs that may exert a protective effect. Our present study found a new method to rescue transplanted CPCs in the infarcted heart by inhibiting CPC apoptosis and autophagy as well as promoting revascularization by treatment with BK, which will provide a novel therapeutic approach for MI.

## Supplementary Information

**Additional file 1: Supplementary Figure 1.** Characteristics of human cardiac c-Kit+ progenitor cells. (A). Representative flow cytometry image of the characteristics of the hCPCs with PE-conjunct CD8A, CD29, CD34, CD45, CD105, CD133, and CD117 antibodies. (B). Representative immunostaining image of hCPCs with an anti-CD117 antibody (marker of c-kit+ cells, red) and DAPI (blue) for nucleus staining. (C). Representative immunostaining image of multipotent-related protein SOX2 and Oct-3/4 of the isolated hCPCs with an anti-SOX2 (green) antibody, an anti-Oct-3/4 (red) antibody, and DAPI (blue) for nucleus staining.

## Data Availability

All data generated or analyzed during this study are included in this published article or are available from the corresponding author upon reasonable request.

## Abbreviations

BK: Bradykinin
hCPCs: Human cardiac c-Kit+ progenitor cells
HF: Heart failure
MI: Myocardial infarction
DCFH-DA: 2′7′-Dichlorofluorescein diacetate
α-MEM: α-Modified Eagle’s medium
FBS: Fetal bovine serum
MTT: 3-(4,5-Dimethyl-2-thiazolyl)-2,5-diphenyl-2-H-tetrazolium bromide
ROS: Reactive oxygen species
TdT: Terminal deoxynucleotidyl transferase
TUNEL: Terminal deoxynucleotidyl transferase-mediated dUTP nick-end labeling
LVEF: Left ventricular ejection fraction
LVFS: Left ventricular shortening fraction
LVEDd: Left ventricular end-diastolic diameter
LVESDd: Left ventricular end-systolic diameter
H&E: Hematoxylin and eosin

## References

[1] Minicucci MF, Azevedo PS, Polegato BF, Paiva SA, Zornoff LA (2011). Heart failure after myocardial infarction: clinical implications and treatment. Clin Cardiol.

[2] Sanganalmath SK, Bolli R. Cell therapy for heart failure: a comprehensive overview of experimental and clinical studies, current challenges, and future directions. Circ Res. 2013;113(6):810–34. 10.1161/CIRCRESAHA.113.300219. 10.1161/CIRCRESAHA.113.300219 PMC3892665 23989721

[3] Bolli R, Tang XL, Guo Y, Li Q. After the storm: an objective appraisal of the efficacy of c-kit+ cardiac progenitor cells in preclinical models of heart disease. Can J Physiol Pharmacol. 2021;99(2):129–39. 10.1139/cjpp-2020-0406. 10.1139/cjpp-2020-0406 PMC8299902 32937086

[4] Balsam LB, Wagers AJ, Christensen JL, Kofidis T, Weissman IL, Robbins RC (2004). Haematopoietic stem cells adopt mature haematopoietic fates in ischaemic myocardium. Nature.

[5] Eschenhagen T, Bolli R, Braun T, Field LJ, Fleischmann BK, Frisen J, Giacca M, Hare JM, Houser S, Lee RT (2017). Cardiomyocyte regeneration: a consensus statement. Circulation.

[6] Murry CE, Soonpaa MH, Reinecke H, Nakajima H, Nakajima HO, Rubart M, Pasumarthi KB, Virag JI, Bartelmez SH, Poppa V (2004). Haematopoietic stem cells do not transdifferentiate into cardiac myocytes in myocardial infarcts. Nature.

[7] Tang XL, Li Q, Rokosh G, Sanganalmath SK, Chen N, Ou Q, Stowers H, Hunt G, Bolli R (2016). Long-term outcome of administration of c-kit(POS) cardiac progenitor cells after acute myocardial infarction: transplanted cells do not become cardiomyocytes, but structural and functional improvement and proliferation of endogenous cells persist for at least one year. Circ Res.

[8] Dergilev K, Tsokolaeva Z, Makarevich P, Beloglazova I, Zubkova E, Boldyreva M, Ratner E, Dyikanov D, Menshikov M, Ovchinnikov A (2018). C-kit cardiac progenitor cell based cell sheet improves vascularization and attenuates cardiac remodeling following myocardial infarction in rats. Biomed Res Int.

[9] Kintsurashvili E, Duka A, Ignjacev I, Pattakos G, Gavras I, Gavras H (2005). Age-related changes of bradykinin B1 and B2 receptors in rat heart. Am J Physiol Heart Circ Physiol.

[10] Fu C, Cao Y, Li B, Xu R, Sun Y, Yao Y (2019). Bradykinin protects cardiac c-kit positive cells from high-glucose-induced senescence through B2 receptor signaling pathway. J Cell Biochem.

[11] Li G, Wang Y, Li GR (2017). Bradykinin regulates cell growth and migration in cultured human cardiac c-Kit+ progenitor cells. Oncotarget.

[12] Li G, Che H, Wu WY, Jie LJ, Xiao GS, Wang Y, Li GR (2018). Bradykinin-mediated Ca(2+) signalling regulates cell growth and mobility in human cardiac c-Kit(+) progenitor cells. J Cell Mol Med.

[13] Zhang YY, Li G, Che H, Sun HY, Li X, Au WK, Xiao GS, Wang Y, Li GR (2014). Characterization of functional ion channels in human cardiac c-kit+ progenitor cells. Basic Res Cardiol.

[14] Beltrami AP, Barlucchi L, Torella D, Baker M, Limana F, Chimenti S, Kasahara H, Rota M, Musso E, Urbanek K, Leri A, Kajstura J, Nadal-Ginard B, Anversa P (2003). Adult cardiac stem cells are multipotent and support myocardial regeneration. Cell.

[15] Che H, Li G, Sun HY, Xiao GS, Wang Y, Li GR (2015). Roles of store-operated Ca2+ channels in regulating cell cycling and migration of human cardiac c-kit+ progenitor cells. Am J Physiol Heart Circ Physiol.

[16] Zhang YY, Li G, Che H, Sun HY, Xiao GS, Wang Y, Li GR (2015). Effects of BKCa and Kir2.1 channels on cell cycling progression and migration in human cardiac c-kit+ progenitor cells. PLoS One.

[17] Che H, Xiao GS, Sun HY, Wang Y, Li GR (2016). Functional TRPV2 and TRPV4 channels in human cardiac c-kit(+) progenitor cells. J Cell Mol Med.

[18] Golpanian S, Schulman IH, Ebert RF, Heldman AW, DiFede DL, Yang PC, Wu JC, Bolli R, Perin EC, Moye L (2016). Concise review: review and perspective of cell dosage and routes of administration from preclinical and clinical studies of stem cell therapy for heart disease. Stem Cells Transl Med.

[19] Fathi E, Valipour B, Vietor I, Farahzadi R (2020). An overview of the myocardial regeneration potential of cardiac c-Kit(+) progenitor cells via PI3K and MAPK signaling pathways. Future Cardiol.

[20] Zhou B, Wu SM (2018). Reassessment of c-Kit in cardiac cells: a complex interplay between expression, fate, and function. Circ Res.

[21] Cai C, Guo Y, Teng L, Nong Y, Tan M, Book MJ, Zhu X, Wang XL, Du J, Wu WJ (2015). Preconditioning human cardiac stem cells with an HO-1 inducer exerts beneficial effects after cell transplantation in the infarcted murine heart. Stem Cells.

[22] Ng KM, Chan YC, Lee YK, Lai WH, Au KW, Fung ML, Siu CW, Li RA, Tse HF (2011). Cobalt chloride pretreatment promotes cardiac differentiation of human embryonic stem cells under atmospheric oxygen level. Cell Reprogram.

[23] Shinmura D, Togashi I, Miyoshi S, Nishiyama N, Hida N, Tsuji H, Tsuruta H, Segawa K, Tsukada Y, Ogawa S, Umezawa A (2011). Pretreatment of human mesenchymal stem cells with pioglitazone improved efficiency of cardiomyogenic transdifferentiation and cardiac function. Stem Cells.

[24] Qi H, Soto-Gonzalez L, Krychtiuk KA, Ruhittel S, Kaun C, Speidl WS, Kiss A, Podesser BK, Yao S, Markstaller K, Klein KU, Tretter V (2018). Pretreatment with argon protects human cardiac myocyte-like progenitor cells from oxygen glucose deprivation-induced cell death by activation of AKT and differential regulation of mapkinases. Shock.

[25] Broughton K, Korski K, Echeagaray O, Adamson R, Dembitsky W, Lu Z, Schaefer E, Sussman MA (2019). Safety profiling of genetically engineered Pim-1 kinase overexpression for oncogenicity risk in human c-kit+ cardiac interstitial cells. Gene Ther.

[26] Zhang Z, Yang J, Yan W, Li Y, Shen Z, Asahara T. Pretreatment of cardiac stem cells with exosomes derived from mesenchymal stem cells enhances myocardial repair. J Am Heart Assoc. 2016;5:e002856. 10.1161/JAHA.115.002856. 10.1161/JAHA.115.002856 PMC4859399 26811168

[27] Leeb-Lundberg LM, Marceau F, Muller-Esterl W, Pettibone DJ, Zuraw BL (2005). International union of pharmacology. XLV. Classification of the kinin receptor family: from molecular mechanisms to pathophysiological consequences. Pharmacol Rev.

[28] Chao J, Shen B, Gao L, Xia CF, Bledsoe G, Chao L (2010). Tissue kallikrein in cardiovascular, cerebrovascular and renal diseases and skin wound healing. Biol Chem.

[29] Chao J, Yin H, Gao L, Hagiwara M, Shen B, Yang ZR, Chao L (2008). Tissue kallikrein elicits cardioprotection by direct kinin b2 receptor activation independent of kinin formation. Hypertension.

[30] Zwetsloot PP, Vegh AM (2016). Jansen of Lorkeers SJ, van Hout GP, Currie GL, Sena ES, Gremmels H, Buikema JW, Goumans MJ, Macleod MR et al: Cardiac stem cell treatment in myocardial infarction: a systematic review and meta-analysis of preclinical studies. Circ Res.

[31] Dumoulin MJ, Adam A, Rouleau JL, Gosselin H, Lamontagne D (2003). Bradykinin metabolism in rat hearts with left-ventricular hypertrophy following myocardial infarction. Can J Physiol Pharmacol.

[32] Ito H, Hayashi I, Izumi T, Majima M (2003). Bradykinin inhibits development of myocardial infarction through B2 receptor signalling by increment of regional blood flow around the ischaemic lesions in rats. Br J Pharmacol.

[33] Fu C, Li B, Sun Y, Ma G, Yao Y (2015). Bradykinin inhibits oxidative stress-induced senescence of endothelial progenitor cells through the B2R/AKT/RB and B2R/EGFR/RB signal pathways. Oncotarget.

[34] Pillat MM, Lameu C, Trujillo CA, Glaser T, Cappellari AR, Negraes PD, Battastini AM, Schwindt TT, Muotri AR, Ulrich H (2016). Bradykinin promotes neuron-generating division of neural progenitor cells through ERK activation. J Cell Sci.

[35] Sheng Z, Yao Y, Li Y, Yan F, Huang J, Ma G (2013). Bradykinin preconditioning improves therapeutic potential of human endothelial progenitor cells in infarcted myocardium. PLoS One.

[36] Sheng ZL, Yao YY, Li YF, Fu C, Ma GS (2015). Transplantation of bradykinin-preconditioned human endothelial progenitor cells improves cardiac function via enhanced Akt/eNOS phosphorylation and angiogenesis. Am J Transl Res.

[37] Park JH, Choi SH, Kim H, Ji ST, Jang WB, Kim JH, et al. Doxorubicin regulates autophagy signals via accumulation of cytosolic Ca(2+) in human cardiac progenitor cells. Int J Mol Sci. 2016;17(10):1680. 10.3390/ijms17101680. 10.3390/ijms17101680 PMC5085713 27735842

[38] Zhang Z, Yang C, Shen M, Yang M, Jin Z, Ding L, Jiang W, Yang J, Chen H, Cao F, Hu T (2017). Autophagy mediates the beneficial effect of hypoxic preconditioning on bone marrow mesenchymal stem cells for the therapy of myocardial infarction. Stem Cell Res Ther.

[39] Ma W, Ding F, Wang X, Huang Q, Zhang L, Bi C, Hua B, Yuan Y, Han Z, Jin M, Liu T, Yu Y, Cai B, du Z (2018). By targeting Atg7 MicroRNA-143 mediates oxidative stress-induced autophagy of c-Kit(+) mouse cardiac progenitor cells. EBioMedicine.

[40] Shi X, Li W, Liu H, Yin D, Zhao J (2017). The ROS/NF-kappaB/NR4A2 pathway is involved in H2O2 induced apoptosis of resident cardiac stem cells via autophagy. Oncotarget.

[41] Lei FR, Li XQ, Liu H, Zhu RD, Meng QY, Rong JJ (2012). Rapamycin and 3-methyladenine regulate apoptosis and autophagy in bone-derived endothelial progenitor cells. Chin Med J (Engl).

[42] Wang C, Mao C, Lou Y, Xu J, Wang Q, Zhang Z, Tang Q, Zhang X, Xu H, Feng Y (2018). Monotropein promotes angiogenesis and inhibits oxidative stress-induced autophagy in endothelial progenitor cells to accelerate wound healing. J Cell Mol Med.

[43] Li X, Hu X, Wang J, Xu W, Yi C, Ma R, Jiang H (2018). Inhibition of autophagy via activation of PI3K/Akt/mTOR pathway contributes to the protection of hesperidin against myocardial ischemia/reperfusion injury. Int J Mol Med.

[44] Liu YY, Sun C, Xue FS, Yang GZ, Li HX, Liu Q, Liao X (2018). Effect of autophagy inhibition on the protection of ischemia preconditioning against myocardial ischemia/reperfusion injury in diabetic rats. Chin Med J (Engl).

[45] Jeong YM, Cheng XW, Lee KH, Lee S, Cho H, Kim W (2020). Substance P enhances the local activation of NK1R-expressing c-kit(+) cardiac progenitor cells in right atrium of ischemia/reperfusion-injured heart. BMC Mol Cell Biol.

