# Supplementary material for: Pretreatment of cardiac progenitor cells with bradykinin attenuates H2O2-induced cell apoptosis and improves cardiac function in rats by regulating autophagy
Source: Stem Cell Res Ther. 2021 Aug 5;12:437. doi: 10.1186/s13287-021-02503-6 (PMC8340370; doi:10.1186/s13287-021-02503-6)
Supplement: Supplementary file 1 — Additional file 1: Supplementary Figure 1. Characteristics of human cardiac c-Kit+ progenitor cells. (A). Representative flow cytometry image of the characteristics of the hCPCs with PE-conjunct CD8A, CD29, CD34, CD45, CD105, CD133, and CD117 antibodies. (B). Representative immunostaining image of hCPCs with an anti-CD117 antibody (marker of c-kit+ cells, red) and DAPI (blue) for nucleus staining. (C). Representative immunostaining image of multipotent-related protein SOX2 and Oct-3/4 of the isolated hCPCs with an anti-SOX2 (green) antibody, an anti-Oct-3/4 (red) antibody, and DAPI (blue) for nucleus staining. [file 13287_2021_2503_MOESM1_ESM.docx]

**Supplemental information**

**
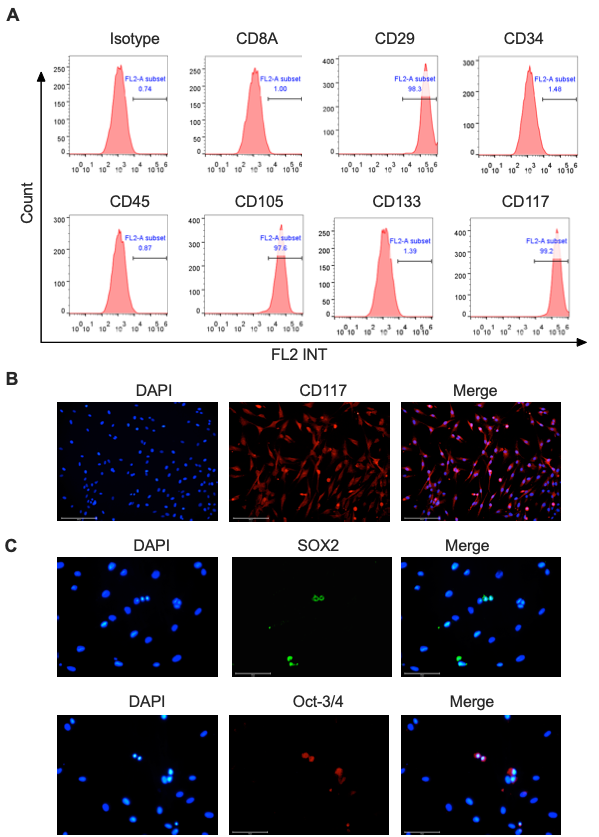
**

**Supplemental figure 1. Characteristics of human cardiac c-Kit+ progenitor cells. (A).** Representative flow cytometry image of the characteristics of the hCPCs with PE-conjunct CD8A, CD29, CD34, CD45, CD105, CD133, and CD117 antibodies. **(B).** Representative immunostaining image of hCPCs with an anti-CD117 antibody (marker of c-kit+ cells, red) and DAPI (blue) for nucleus staining. **(C)**. Representative immunostaining image of multipotent-related protein SOX2 and Oct-3/4 of the isolated hCPCs with an anti-SOX2 (green) antibody and an anti-Oct-3/4 (red) antibody and DAPI (blue) for nucleus staining.
